# Estimation of years lived with disability due to noncommunicable diseases and injuries using a population-representative survey

**DOI:** 10.1371/journal.pone.0172001

**Published:** 2017-02-14

**Authors:** Ji In Park, Hae Hyuk Jung

**Affiliations:** Department of Medicine, Kangwon National University Hospital, Kangwon National University School of Medicine, Chuncheon, Gangwon-do, South Korea; University of Auckland, NEW ZEALAND

## Abstract

The Global Burden of Disease 2010 and the WHO Global Health Estimates of years lived with disability (YLDs) uses disability-weights obtained from lay health-state descriptions, which cannot fully reflect different disease manifestations, according to severity, treatment, and environment. The aim of this study was to provide population-representative YLDs of noncommunicable diseases and injuries using a prevalence-based approach, with the disability weight measured in subjects with specific diseases or injuries. We included a total of 44969 adults, who completed the EQ-5D questionnaire as participation in the Korea National Health and Nutrition Examination Survey 2007–2014. We estimated the prevalence of each of 40 conditions identified from the noncommunicable diseases and injuries in the WHO list. Modified condition-specific disability-weight was determined from the adjusted mean difference of the EQ-5D index between the condition and reference groups. Condition-specific YLDs were calculated as the condition’s prevalence multiplied by the condition’s disability-weight. All-cause YLDs, estimated as “number of population × (1 − mean score of EQ-5D)” were 2165 thousands in 39044 thousand adults aged ≥20. The combined YLDs for all 40 conditions accounted for 67.6% of all-cause YLDs, and were 1604, 2126, 8749, and 12847 per 100000 young (age 20−59) males, young females, old (age ≥60) males, and old females, respectively. Back pain/osteoarthritis YLDs were exceptionally large (442/40, 864/146, 2037/836, and 4644/3039 per 100000 young males, young females, old males, and old females, respectively). Back pain, osteoarthritis, depression, diabetes, periodontitis, and stroke accounted for 22.3%, 9.1%, 4.6%, 3.3%, 3.2%, and 2.9% of all-cause YLDs, respectively. In conclusion, this estimation of YLDs using prevalence rates and disability-weights measured in a population-representative survey may form the basis for population-level strategies to prevent age-related worsening of disability.

## Introduction

The World Health Organization (WHO) Global Health Estimates (GHE) and World Bank-commissioned Global Burden of Disease (GBD) study measure the overall burden of disease using disability-adjusted life years (DALYs) [1]. This time-based measure combines years of life lost due to premature mortality (YLLs) and years lost due to time lived in states of less than full health (years lived with disability [YLDs]). YLDs are determined by non-fatal health outcomes of diseases and injuries; chronic noncommunicable diseases and injuries with lifelong consequences contribute markedly to non-fatal burdens of disease.

Condition-specific YLDs can be computed as the prevalence of disease or injury multiplied by the disability-weight for that condition [2,3], and their reliable quantification requires precise estimates of prevalence rates and disability-weights for those conditions. However, unfortunately, the epidemiological data currently available have limitations, including lack of information on severity distributions, inconsistent methods for measuring disability-weights, and wide variation in data sources, for most conditions [4–8]. The GBD 2010 employed lay descriptions of the consequences of various diseases and injuries for developing universal measures of disability-weights, distinct from welfare and environments [9]. The WHO GHE also used the GBD 2010-developed disability-weights to calculate global and regional YLDs, after partially revising the values. However, it is arguable whether health and welfare can be separated and whether a universal approach is possible or even desirable [10]. Additionally, a brief lay description cannot reflect various manifestations of the same disease, the effect of treatment on disability, and adaptation to environments.

The Korea National Health and Nutrition Examination Survey (KNHANES) can facilitate estimation of condition-specific YLDs for noncommunicable diseases or injuries in the general population. This large population-representative survey, conducted by the Korea Centers for Disease Control and Prevention (KCDC), used the EQ-5D questionnaire to measure health-related quality of life. The EQ-5D provides a simple descriptive profile and a single index value for health status, simplifying disability-weight calculation. The survey also includes health questionnaires and physical/laboratory examinations, allowing determination of prevalence rate and disability-weight for specific diseases and injuries. Thus, numerous condition-specific YLDs could be estimated from a single source.

The present study aimed to provide population-representative YLDs of noncommunicable diseases and injuries, based on the KNHANES data. Additionally, we compared the YLDs of this study to those of the GHE.

## Materials and methods

### Subjects and identification of conditions

The KNHANES is a population-based, cross-sectional study on the health and nutritional status of the non-institutionalized Korean population. The KCDC conducted the survey using a stratified, multistage, clustered probability design to select a representative, nationwide sample [11]. KNHANES comprises a health questionnaire, physical/laboratory examinations, and a nutrition survey; to date, phase I (1998), II (2001), III (2005), IV (2007−2009), V (2010−2012), and VI (2013−2015) have been executed by the Korean government. Written informed consent was obtained from each participant in the KNHANES at enrollment.

The present study protocol was approved by Kangwon national university hospital institutional review board (IRB File No.: KNUH-2015-06-001). This study was based on KNHANES 2007−2014 data, as these surveys were conducted by a single organization, KCDC, using consistent methodology since 2007, and KNHANES 2015 data were not available at the time of this analysis.

Health-related quality of life was assessed using the Korean version of the EQ-5D health questionnaire. The EQ-5D comprises five dimensions: mobility, self-care, usual activities, pain/discomfort, and anxiety/depression. Each dimension comprised three levels: no problems, moderate problems, and extreme problems. The combination of all dimensions and levels yields 243 unique health states. The EQ-5D index scores were calculated based on the Korean value set, which has been established based on a representative national sample using the time−trade-off method [12]. Scores of 1 and 0 correspond to optimal and worst health, judged to be equivalent to death, respectively.

**Fig 1:**
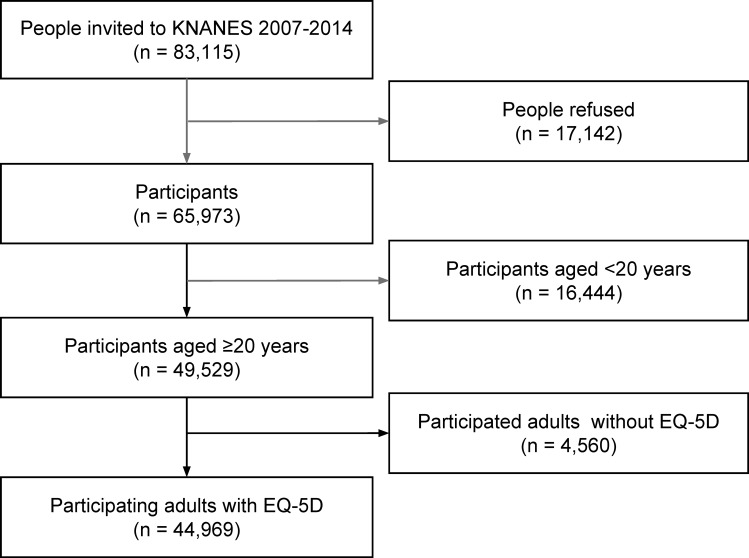
Flow chart of the study participants.

Of the 65973 subjects participating in KNHANES 2007−2014, we excluded subjects younger than 20-years-old (n = 16444) and adults who did not complete the EQ-5D questionnaire (n = 4560). Thus, a total of 44969 subjects (18984 males and 25985 females) were included in this study (Fig 1).

### Physical and laboratory examinations

Trained medical staff and medical specialists, including ophthalmologists, otolaryngologists, and dentists performed the physical examinations, following standardized procedures. Oral health examinations were conducted in mobile centers that traveled to each survey location. Dentists conducted the examinations with the participant seated in a dental chair. Before the oral examination, participants were informed about the procedures using intra-oral pictures, tooth models, and simulation patients. Pulmonary function tests were performed in participants aged ≥ 40 years, using dry rolling seal spirometers. The procedure was conducted by trained medical personnel, who underwent education sessions on pulmonary function tests and quality control prior to the study.

From July 2008 to December 2012, ophthalmological and otological examinations were conducted in the Korea National Health and Nutrition Examination Survey (KNHANES). These examinations were conducted by trained teams from mobile centers; use of such centers provided a standardized environment and equipment. Presenting visual acuity was measured using currently available refractive correction, if any, with an international standard vision chart based on the Snellen scale. Best-corrected visual acuity was measured using autorefraction and/or a pinhole. Details of ophthalmologic examinations, including autorefractometry, slit lamp biomicroscopy, fundus photography, intraocular pressure, and visual field (frequency-doubling technology) tests have been published elsewhere [13]. Each fundus image was reviewed twice: onsite by ophthalmologists or ophthalmological residents, and then by retina specialists. An audiometry test was conducted by well-trained examiners, and the air-conduction hearing threshold was measured in a soundproof booth using an automatic audiometer at 500, 1000, 2000, 3000, 4000, and 6000 Hz.

Since 2010, plain radiographs of the knee, hip, and lumbar spine have been obtained in participants aged ≥ 50 years. The radiographic images were reviewed by two radiologists. The degree of radiographic osteoarthritis was assessed according to the Kellgren−Lawrence grading system.

Blood samples were collected after at least an 8-h fast, and random spot urine samples were obtained. The samples were processed appropriately, immediately refrigerated, and transported in cold storage to the central laboratory within 24 h. Blood hemoglobin and routine chemistries, including glucose and creatinine levels, were analyzed using standard methods. From 2007 to 2012, serum ferritin levels were measured by immunoradiometric assay. Since 2008, serum creatinine levels have been standardized to isotope dilution mass spectrometry, and urine albumin levels have been measured by turbidimetric immunoassay since 2011.

### Identification of conditions

We identified noncommunicable diseases and injuries from the cause list of the World Health Organization (WHO) Global Health Estimates (GHE).[2] The GHE list provides a set of mutually exclusive and collectively exhaustive categories. The causes in the list are categorized into three broad groups: (I) communicable, maternal, perinatal, and nutritional conditions; (II) noncommunicable diseases; and (III) injuries. Among a total of 79 individual diseases of group II, we selected 30 diseases that could be identified using information available from the KNHANES data. Those 30 diseases accounted for two-thirds of the total YLDs related to group II. Nine of 10 individual injuries of group III could be identified, and those accounted for nearly 100% of the total YLDs related to group III. Additionally, we selected iron-deficiency anemia, which accounted for a fourth of the total YLDs related to group I. Of the 40 identified conditions, five were divided into subcategories. Table 1 shows the study years and number of subjects analyzed for each condition.

**Table 1:** Study years and number of subjects analyzed for each condition.

Condition	No. analyzed	2007	2008	2009	2010	2011	2012	2013	2014
Asthma, Cancers, Cirrhosis, Depression, Dermatitis, Ischemic heart disease, Rheumatoid arthritis, Stroke	44,967−44,969	√	√	√	√	√	√	√	√
Iron-deficiency anemia	33,089	√	√	√	√	√	√		
Diabetes mellitus	41,392	√	√	√	√	√	√	√	√
Alcohol-use disorders	33,969	√	√	√	√	√	√	√	
Visual impairment	25,884−28,127		√	√	√	√	√		
Hearing impairment	22,889		√	√	√	√	√	√	
Chronic obstructive pulmonary disease (Age ≥ 40 years)	21,425	√	√	√	√	√	√	√	√
Chronic kidney disease	19,166					√	√	√	√
Peptic ulcer	17,069	√	√	√					
Osteoarthritis, Back pain (Age ≥ 50 years)	11,559				√	√	√	√	
Back pain (Age 20−49 years)	8,955	√	√	√					
Dental caries, Periodontal disease	20,154				√	√	√	√	√
Edentulism	43,536	√	√	√	√	√	√	√	√
Injuries	44,955	√	√	√	√	√	√	√	√

### Definition of diseases and injuries

We defined and classified iron-deficiency anemia according to the WHO criteria [14]. Diabetes mellitus was defined as fasting blood glucose levels ≥ 126 mg/dl, being on medication for raised blood glucose, or with a history of diagnosis of diabetes. Alcohol-use disorders were defined based on the Alcohol-Use Disorders Identification Test scores according to the WHO guidelines [15].

We defined glaucoma according to the criteria of the International Society of Geographical and Epidemiological Ophthalmology classification scheme: Category 1, the presence of reliable (fixation and false-positive error ≤ 1) abnormal visual field testing (≥ one location of reduced sensitivity) plus a vertical cup-to-disc ratio (VCDR) ≥ 0.7, or asymmetry of the VCDR ≥ 0.2, or the presence of optic disk hemorrhage, or a retinal nerve fiber layer defect; Category 2, a VCDR ≥ 0.9 or asymmetry of the VCDR ≥ 0.3, or the presence of a retinal nerve fiber layer defect with violation of the inferior−superior−nasal−temporal rule; or Category 3, an intraocular pressure ≥ 22 mmHg plus a visual acuity < 3/60. Cataract was defined as nuclear (Lens Opacities Classification System [LOCS] III score ≥ 4 for nuclear opalescence or nuclear color), cortical (LOCS III score ≥ 2 for cortical cataracts), posterior subcapsular (LOCS III score ≥ 2 for posterior subcapsular), or mixed (more than one type per eye) based on comparison with standard photographs. The diagnoses of diabetic retinopathy and age-related macular degeneration were made by retina specialists based on fundus photography images using protocols from the Early Treatment for Diabetic Retinopathy Study and International Age-related Maculopathy Epidemiological Study Group. We defined visual impairment as a visual acuity < 6/18 in the better eye, including uncorrected refractive errors according to the International Classification of Diseases-10.

Disabling hearing impairment was defined as an audiometric International Society of Otolaryngology value (average of values at 500, 1000, 2000, 4000 Hz) ≥ 41 decibels in the better ear, according to the WHO classification [16]. Chronic obstructive pulmonary disease was defined and classified based on pulmonary function test results according to the Global Initiative for Chronic Obstructive Lung Disease [17]. Chronic kidney disease was classified into risk categories according to the Kidney Disease Improving Global Outcomes guidelines [18]. Osteoarthritis was defined as the presence of knee or hip pain with Kellgren−Lawrence grading scales score ≥ 2 on the corresponding radiographic images. We assessed periodontitis using the WHO Community Periodontal Index [19]. Periodontal disease was defined as a Community Periodontal Index score ≥ 3 with symptoms of difficulty in chewing and recent toothache.

We defined cancers, depression, ischemic heart disease (myocardial infarction or angina), stroke, current asthma, current peptic ulcer, cirrhosis, atopic dermatitis, and rheumatoid arthritis as a physician-based diagnosis of each disease. Unintentional or intentional injuries were defined based on self-reported questionnaires.

We summarized the definition of each condition in Table 2.

**Table 2:** Definition of noncommunicable diseases and injuries.

GHE code	GHE disease	KNHANES disease	Definition
0	All Causes	All Causes	The number of the population multiplied by the mean score of the combined disability weight (= “1 − mean score of EQ-5D index”)
58	Iron-deficiency anemia	Iron-deficiency anemia	
		Mild	Hb < 13.0 g/dL in men or Hb < 12.0 g/dL in non-pregnant women or Hb <11.0 g/dL in pregnant women, with serum ferritin <15 ug/L
		Moderate	Hb 8.0−10.9 g/dL in men and non-pregnant women or Hb 7.0−9.9 g/dL in pregnant women, with serum ferritin <15 ug/L
		Severe	Hb <8.0 g/dL in men and non-pregnant women or Hb <7.0 g/dL in pregnant women, with serum ferritin <15 ug/L
64	Stomach cancer	Stomach cancer	Physician diagnosed
65	Colon and rectum cancers	Colon cancers	Physician diagnosed
66	Liver cancer	Liver cancer	Physician diagnosed
68	Trachea, bronchus, lung cancers	Lung cancers	Physician diagnosed
70	Breast cancer	Breast cancer	Physician diagnosed
71	Cervix uteri cancer	Cervix cancer	Physician diagnosed
78	Other malignant neoplasms	Other malignancy	Physician diagnosed
80	Diabetes mellitus	Diabetes mellitus	Fasting blood glucose ≥ 7.0 mmol/L (≥ 126 mg/dL) or on medication for raised blood glucose or with a history of diagnosis of diabetes
83	Unipolar depressive disorders	Depression	Physician diagnosed
86	Alcohol-use disorders	Alcohol-use disorders	
		Harmful drinking behavior	Questionnaire-based AUDIT score 16−19
		Alcohol dependence	Questionnaire-based AUDIT score ≥ 20
103	Glaucoma	Glaucoma with visual impairment	Intraocular pressure, fundus photography, and visual field test-based diagnosis of glaucoma with BCVA < 6/18 in the better eye
104	Cataracts	Cataracts with visual impairment	Slit lamp biomicroscopy-based diagnosis of cataract with BCVA < 6/18 in the better eye
105	Refractive errors	Uncorrected refractive errors	Autorefractometer-based diagnosis of refractive errors with presenting VA < 6/18 and BCVA ≥ 6/18 in the better eye
106	Macular degeneration	Macular degeneration with visual impairment	Fundus photography-based diagnosis of macular degeneration with BCVA < 6/18 in the better eye
107	Other vision loss	Diabetic retinopathy with visual impairment	Fundus photography-based diagnosis of diabetic retinopathy with BCVA < 6/18 in the better eye
108	Other hearing loss	Disabling hearing impairment	Audiometric International Society of Otolaryngology value ≥ 41 decibels in the better ear
113	Ischemic heart disease	Ischemic heart disease	Physician diagnosed
114	Stroke	Stroke	Physician diagnosed
118	COPD	COPD, age ≥ 40 years	
		Mild	FEV1/FVC < 70% and FEV1 ≥ 80% predicted
		Moderate	FEV1/FVC < 70% and FEV1 50−79% predicted
		Severe	FEV1/FVC < 70% and FEV1 < 50% predicted
119	Asthma	Asthma	Physician diagnosed, current asthma
122	Peptic ulcer disease	Peptic ulcer, gastric or duodenal	Physician diagnosed, current peptic ulcer
123	Cirrhosis of the liver	Cirrhosis of the liver	Physician diagnosed
127	Kidney diseases	Chronic kidney disease	
		Moderately increased risk	eGFR < 60 mL/min/1.73 m^2^ or ACR ≥ 3 mg/mmol (≥30 mg/g), excluding high risk or very high risk
		High risk	eGFR < 45 mL/min/1.73 m^2^ or ACR ≥ 30 mg/mmol (≥ 300 mg/g) or eGFR < 60 mL/min/1.73 m^2^ with ACR ≥ 3 mg/mmol (≥ 30 mg/g), excluding very high risk
		Very high risk	eGFR < 30 mL/min/1.73 m^2^ or eGFR < 45 mL/min/1.73 m^2^ with ACR ≥ 3 mg/mmol (≥ 30 mg/g) or eGFR <60 mL/min/1.73 m^2^ with ACR ≥ 30 mg/mmol (≥ 300 mg/g)
133	Skin diseases	Atopic dermatitis	Physician diagnosed
135	Rheumatoid arthritis	Rheumatoid arthritis	Physician diagnosed
136	Osteoarthritis	OA, age ≥50 years	Knee pain with K-L grading scale ≥ 2 on knee X-ray or hip pain with K-L grading scale ≥ 2 on hip X-ray
138	Back and neck pain	Back pain	
		Back pain with radiographic OA, age ≥50 years	Recent (within 3 months) back pain with duration of ≥ 1 month, with K-L grading scale ≥ 2 on lumbar spine X-ray
		Back pain without radiographic OA, age ≥50 years	Recent (within 3 months) back pain with duration of ≥1 month, with K-L grading scale < 2 on L-spine X-ray
		Back pain, age 20−49 years	Physician diagnosed, current back pain
148	Dental caries	Dental caries	Dental exam-based diagnosis of caries with difficulty chewing and recent (within 1 year) toothache
149	Periodontal disease	Periodontal disease	Dental exam-based diagnosis of periodontal disease with difficulty chewing and recent (within 1 year) toothache
150	Edentulism	Edentulism	Dental exam-based diagnosis of severe tooth loss needing full dentures
153	Road injury	Road injury	Self-reported, recent (within 1 year) road injury
154	Poisonings	Poisonings	Self-reported, recent (within 1 year) poisonings
155	Falls	Falls	Self-reported, recent (within 1 year) falls
156	Fire, heat, and hot substances	Fire and heat injury	Self-reported, recent (within 1 year) fire and heat injury
157	Drowning	Drowning	Self-reported, recent (within 1 year) drowning
158	Exposure to forces of nature	Injuries from other mechanical forces	Self-reported, recent (within 1 year) injuries from other mechanical forces
159	Other unintentional injuries	Other unintentional injuries	Self-reported, recent (within 1 year) other unintentional injuries
161	Self-harm	Self-harm	Self-reported, recent (within 1 year) self-harm
162	Interpersonal violence	Violence	Self-reported, recent (within 1 year) interpersonal violence

Abbreviations: GHE, Global Health Estimates; KNHANES, Korea National Health and Nutrition Examination Survey; Hb, hemoglobin; AUDIT, alcohol-use disorders identification test; VA, visual acuity; BCVA, best-corrected visual acuity; COPD, chronic obstructive pulmonary disease; FEV1, forced expiratory volume 1; FVC, forced vital capacity; eGFR, estimated glomerular filtration rate; ACR, urinary albumin creatinine ratio; K-L grading scale, Kellgren−Lawrence grading scale; OA, osteoarthritis.

Physician-diagnosed diseases were defined according to self-reported medical history; most diseases according to the response to “diagnosed by a physician” of the health questionnaire, whereas both the current asthma and current peptic ulcer according to the response to “currently having the disease”.

### Statistical analysis and computation of disability-weights and YLDs

Statistical analyses were performed with SPSS (version 22.0). Since the KCDC conducted the KNHANES using a complex survey design, we used SPSS Complex Samples modules to produce reliable point estimates and robust standard errors.

We computed YLDs as follows. First, we estimated the prevalence of each condition in every 2 × 2 age−sex group, arranged according to an age cutoff of 60 years and sex, as well as in the total population. Composite sample weights were introduced separately in each of the analyses to provide representative estimates of the Korean population. We calculated a total of nine composite sample weights by multiplying the survey sample weights by the year weights, according to KCDC’s guidebook. The survey sample weight for each examination was computed using the sampling rate, response rate, and age−sex proportion of the Korean population. The year weight for each examination was determined by the number of households that participated in that year’s examination.

Second, a general linear model was used to test the effect of each condition on the EQ-5D index, introducing the composite sample weight. Each adjusted mean difference from the reference group was computed using the estimated marginal means of the EQ-5D index; these were estimated as the mean value, averaged over all cells generated by the age (and sex for the analysis of the total population) category, after subdividing age into seven 10-year-width categories, from 20−29 years through to 70−79 years, as well as ≥ 80 years. For comparison, all other subjects without each condition served as the reference group. The differences in these age (and sex)-adjusted mean scores between the condition and reference groups were obtained for each of the 2 × 2 age−sex groups, as well as for the total population. As in the GBD 2010 and GHE [2,3], it was assumed that the conditions co-occurred independently of each other within the age (and sex) category. However, for osteoarthritis and back pain (as well as caries and periodontitis), which were most prevalent and which were substantially correlated with each other, both conditions were introduced into the model together, to adjust for the effect of dependence. If the two-tailed *P* value exceeded 0.10 (one-tailed *P* value > 0.05), the value of the adjusted mean difference was excluded from further analyses.

Third, the “adjusted mean difference of the EQ-5D index” was used to establish a condition-specific disability-weight. To estimate the comorbidity-adjusted effect of each condition on disability (“*condition specific DW*”), the disability-weight that included the condition of interest (“*combined DW*”) was compared with the disability-weight that excluded the condition of interest (“*comorbid DW*”). Our approach was comparable to that of the GBD 2010 and GHE. Assuming that comorbid conditions change the quantitative score for the health-related quality of life multiplicatively rather than additively,

1−(comorbidDW)×(1−conditionspecificDW)=(1−combinedDW)

2−(1−comorbidDW)−(1−combinedDW)=conditionspecificDW×(1−comorbidDW)

Assuming that comorbidities were independently distributed in the condition and reference groups within the age (and sex) category, the value of “1 – *comorbid DW*” was replaced with the estimated “marginal mean of EQ-5D index in the reference group”, and the value of “1 – *combined DW*” was replaced with the “marginal mean in the group with the condition of interest”.

We computed YLDs as the prevalence of each condition multiplied by the condition’s disability-weight; this prevalence-based method had also been used in both the GBD 2010 and GHE:

3−YLD=prevalence×DW

4−comorbiditycorrectedconditionspecificYLD=combinedYLD−comorbidYLD=prevalence×(combinedDW−comorbidDW)

The value of “*combined DW – comorbid DW*” was replaced with the “adjusted mean difference of the EQ-5D index” between the condition and reference groups in our study. The same value had been calculated from “*condition specific DW* × (1 – *comorbid DW*)” in the GBD 2010 and GHE [2,3].

All-cause YLDs were estimated as the number of the population multiplied by the mean score of the combined disability-weight (= “1 – *mean score of EQ*5*D index*”).

We used the Korean population count released by Statistics Korea for 2012 in computing YLDs. Our YLDs were then compared with those of the WHO GHE 2014, the WHO’s most recent update of the GHE for 2012.

## Results

### Subject characteristics and EQ-5D index

**Fig 2:**
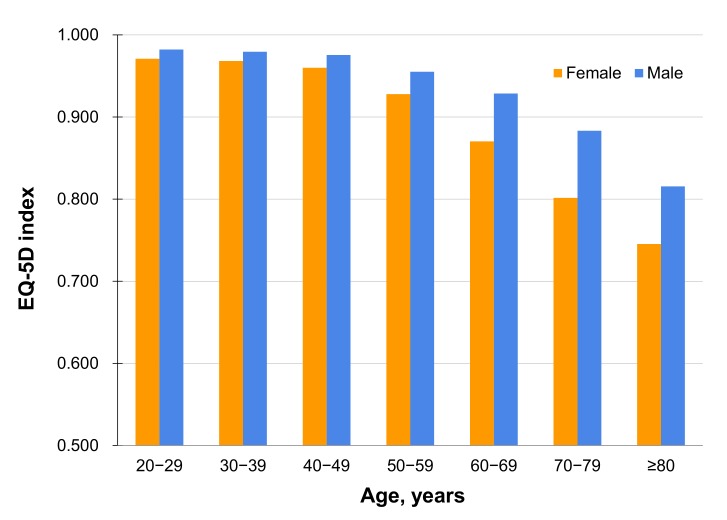
EQ-5D index according to age and sex.

The unweighted/weighted mean age of the study population was 50.4/45.6 years, and 57.8%/50.7% were female. The mean score of unweighted EQ-5D index of the study sample was 0.930, and the mean score of weighted EQ-5D index for the Korean population was 0.945. The mean score of the EQ-5D index decreased with age, and the age-related decrease in EQ-5D was more marked in females (Fig 2).

### Prevalence and modified disability-weight

Table 3 shows the prevalence rates and the modified condition-specific disability-weights for noncommunicable diseases and injuries. Diabetes, alcohol-use disorders, hearing impairment, chronic obstructive pulmonary disease (COPD), chronic kidney disease (CKD), osteoarthritis, back pain, and periodontitis were common conditions, with a prevalence ≥ 5%. Iron-deficiency anemia was most prevalent in young females. Depression was more prevalent in females than in males. Alcohol-use disorders, road injury, other mechanical injuries, other unintentional injuries, and violence were most prevalent in young males. The prevalence rates of diabetes, visual/hearing impairment, ischemic heart disease, stroke, COPD, CKD, osteoarthritis, back pain, periodontitis, and edentulism were markedly higher in old than in young people. Osteoarthritis and back pain were very common in old females. Dental caries was common in all of the age−sex groups.

**Table 3:** The prevalence rates and the modified disability weights for noncommunicable diseases and injuries.

		Prevalence estimate, %					Disability weight (SE)		*P*^a^	Disability weight (SE)		*P*^a^	Disability weight (SE)		*P*^a^	Disability weight (SE)		*P*^a^	Disability weight (SE)		*P*^a^
GHE code		Total	Age 20−59 years		Age ≥ 60 years		Total			Age 20−59 years						Age ≥ 60 years					
	KNHANES disease		Male	Female	Male	Female				Male			Female			Male			Female		
0	All Causes						0.055	(0.001)		0.026	(0.001)		0.043	(0.001)		0.095	(0.002)		0.171	(0.003)	
58	Iron-deficiency anemia																				
	Mild	2.18%	0.20%	4.81%	0.98%	1.18%	-0.009	(0.003)	.003	0.014	(0.016)	.402	-0.005	(0.003)	.060	-0.014	(0.021)	.506	0.011	(0.026)	.684
	Moderate	2.44%	0.19%	4.90%	1.13%	3.25%	0.008	(0.005)	.146	0.042	(0.020)	.032	0.005	(0.005)	.374	0.103	(0.041)	.012	0.014	(0.015)	.362
	Severe	0.19%	0.03%	0.36%	0.12%	0.21%	0.015	(0.014)	.296	-0.007	(0.021)	.740	0.007	(0.016)	.658	0.115	(0.066)	.083	0.066	(0.048)	.173
64	Stomach cancer	0.54%	0.24%	0.21%	2.55%	1.16%	-0.001	(0.009)	.908	-0.013	(0.010)	.207	0.013	(0.020)	.523	0.009	(0.015)	.559	0.005	(0.021)	.793
65	Colon cancers	0.30%	0.18%	0.06%	1.49%	0.66%	0.011	(0.014)	.430	-0.009	(0.012)	.449	-0.011	(0.028)	.701	0.045	(0.026)	.087	0.003	(0.030)	.915
66	Liver cancer	0.10%	0.11%	0.02%	0.42%	0.07%	0.008	(0.022)	.720	-0.024	(0.009)	.005	0.150	(0.039)	.000	0.025	(0.049)	.610	0.019	(0.027)	.467
68	Lung cancers	0.07%	0.03%	0.02%	0.43%	0.08%	0.041	(0.035)	.249	0.061	(0.024)	.013	-0.031	(0.036)	.391	0.098	(0.059)	.099	-0.084	(0.028)	.003
70	Breast cancer	0.35%	0.00%	0.58%	0.00%	1.09%	0.035	(0.015)	.022				0.031	(0.015)	.039				0.028	(0.033)	.388
71	Cervix cancer	0.37%	0.00%	0.49%	0.00%	1.55%	-0.004	(0.009)	.640				0.005	(0.009)	.605				-0.028	(0.015)	.071
78	Other malignancy	1.11%	0.43%	1.23%	2.65%	1.89%	0.002	(0.006)	.689	-0.004	(0.009)	.693	0.004	(0.007)	.557	0.010	(0.015)	.507	0.010	(0.019)	.604
80	Diabetes mellitus	8.71%	7.09%	4.11%	23.00%	20.73%	0.020	(0.003)	.000	0.015	(0.004)	.000	0.021	(0.006)	.000	0.026	(0.006)	.000	0.021	(0.007)	.002
83	Depression	3.69%	1.71%	5.01%	2.49%	7.07%	0.072	(0.005)	.000	0.100	(0.015)	.000	0.065	(0.005)	.000	0.075	(0.016)	.000	0.060	(0.009)	.000
86	Alcohol use disorders																				
	Harmful drinking behavior	6.81%	12.11%	2.12%	6.69%	0.26%	0.004	(0.002)	.094	0.002	(0.002)	.369	0.015	(0.007)	.034	-0.011	(0.009)	.262	-0.009	(0.050)	.854
	Alcohol dependence	6.91%	11.95%	2.40%	7.22%	0.43%	0.017	(0.003)	.000	0.013	(0.003)	.000	0.033	(0.010)	.001	0.016	(0.010)	.099	0.056	(0.039)	.150
103	Glaucoma with visual impairment	0.21%	0.11%	0.06%	0.55%	0.79%	0.028	(0.030)	.338	0.040	(0.022)	.074	0.010	(0.023)	.672	0.061	(0.046)	.192	0.007	(0.065)	.915
104	Cataracts with visual impairment	1.28%	0.31%	0.22%	4.26%	6.21%	0.059	(0.012)	.000	0.051	(0.016)	.001	0.050	(0.023)	.028	0.093	(0.023)	.000	0.039	(0.017)	.026
105	Uncorrected refractive errors	3.85%	2.47%	4.26%	4.10%	7.27%	0.011	(0.005)	.015	0.022	(0.007)	.003	0.009	(0.006)	.097	-0.019	(0.015)	.201	0.014	(0.014)	.312
106	Macular degeneration with visual impairment	0.17%	0.03%	0.01%	0.88%	0.86%	0.079	(0.038)	.039	-0.043	(0.003)	.000	0.015	(0.002)	.000	0.100	(0.067)	.137	0.087	(0.045)	.053
107	Diabetic retinopathy with visual impairment	0.07%	0.01%	0.03%	0.23%	0.37%	0.071	(0.032)	.027	0.086	(0.002)	.000	-0.021	(0.038)	.587	0.124	(0.074)	.093	0.060	(0.038)	.111
108	Disabling hearing impairment	6.92%	3.36%	2.68%	21.42%	18.69%	0.014	(0.005)	.002	0.008	(0.007)	.246	0.001	(0.006)	.875	0.032	(0.008)	.000	0.018	(0.011)	.097
113	Ischemic heart disease	1.80%	0.87%	0.58%	6.71%	5.43%	0.052	(0.007)	.000	0.057	(0.018)	.000	0.041	(0.011)	.000	0.040	(0.011)	.001	0.068	(0.013)	.000
114	Stroke	1.54%	0.68%	0.42%	6.31%	4.69%	0.102	(0.009)	.000	0.114	(0.022)	.000	0.075	(0.023)	.001	0.117	(0.013)	.000	0.096	(0.016)	.000
118	COPD, age ≥40 years																				
	Mild	6.23%	4.38%	1.11%	20.56%	7.31%	-0.003	(0.005)	.502	0.007	(0.007)	.336	-0.004	(0.011)	.686	0.000	(0.007)	.976	0.013	(0.013)	.328
	Moderate	6.55%	5.59%	1.75%	19.93%	6.23%	0.003	(0.004)	.455	0.011	(0.007)	.124	0.026	(0.010)	.010	0.009	(0.006)	.151	0.003	(0.015)	.863
	Severe	0.68%	0.50%	0.21%	2.48%	0.47%	0.030	(0.015)	.043	0.062	(0.029)	.033	0.092	(0.072)	.202	0.034	(0.017)	.042	0.002	(0.041)	.969
119	Asthma	1.57%	0.94%	1.10%	2.88%	4.37%	0.061	(0.007)	.000	0.037	(0.013)	.004	0.044	(0.013)	.001	0.076	(0.016)	.000	0.083	(0.016)	.000
122	Peptic ulcer, gastric or duodenal	1.05%	0.82%	0.81%	1.73%	2.31%	0.077	(0.013)	.000	0.061	(0.023)	.007	0.056	(0.019)	.003	0.073	(0.043)	.092	0.119	(0.030)	.000
123	Cirrhosis of the liver	0.23%	0.22%	0.10%	0.68%	0.33%	0.012	(0.011)	.278	0.009	(0.017)	.618	0.021	(0.028)	.464	0.033	(0.024)	.162	-0.003	(0.022)	.890
127	Chronic kidney disease																				
	Moderately increased risk	7.20%	4.08%	4.72%	17.23%	18.46%	0.010	(0.004)	.011	0.010	(0.007)	.132	0.002	(0.005)	.758	0.030	(0.010)	.002	0.005	(0.009)	.582
	High risk	1.33%	0.63%	0.51%	4.97%	3.57%	0.019	(0.010)	.072	0.019	(0.019)	.315	0.029	(0.018)	.103	0.014	(0.013)	.285	0.034	(0.025)	.179
	Very high risk	0.49%	0.25%	0.20%	1.58%	1.45%	0.074	(0.018)	.000	0.092	(0.042)	.030	0.028	(0.017)	.112	0.079	(0.027)	.003	0.088	(0.035)	.011
133	Atopic dermatitis	2.79%	3.15%	3.33%	1.16%	0.94%	0.013	(0.003)	.000	0.010	(0.004)	.012	0.009	(0.004)	.037	0.047	(0.021)	.028	0.086	(0.032)	.007
135	Rheumatoid arthritis	1.66%	0.65%	1.56%	1.67%	5.56%	0.069	(0.008)	.000	0.060	(0.014)	.000	0.055	(0.011)	.000	0.085	(0.023)	.000	0.072	(0.014)	.000
136	OA, age ≥50 years	12.84%	2.43%	7.65%	8.16%	28.86%	0.099	(0.006)	.000	0.070	(0.020)	.000	0.077	(0.011)	.000	0.102	(0.012)	.000	0.105	(0.009)	.000
138	Back pain																				
	Back pain with radiographic OA, age ≥50 years	10.37%	2.66%	4.77%	7.80%	22.94%	0.125	(0.008)	.000	0.132	(0.033)	.000	0.104	(0.013)	.000	0.122	(0.014)	.000	0.129	(0.010)	.000
	Back pain without radiographic OA, age ≥50 years	12.39%	7.39%	14.88%	8.82%	17.01%	0.091	(0.006)	.000	0.064	(0.012)	.000	0.078	(0.009)	.000	0.123	(0.014)	.000	0.099	(0.009)	.000
	Back pain, age 20−49 years	8.53%	6.45%	10.73%			0.054	(0.005)	.000	0.050	(0.008)	.000	0.056	(0.006)	.000						
148	Dental caries	4.94%	5.05%	3.88%	7.44%	6.13%	0.022	(0.005)	.000	0.012	(0.007)	.075	0.023	(0.008)	.002	0.037	(0.018)	.035	0.047	(0.021)	.026
149	Periodontal disease	5.34%	5.36%	2.51%	11.98%	10.42%	0.033	(0.006)	.000	0.013	(0.007)	.058	0.033	(0.010)	.001	0.033	(0.016)	.039	0.064	(0.018)	.000
150	Edentulism	2.20%	0.36%	0.17%	8.76%	10.17%	0.027	(0.007)	.000	-0.002	(0.013)	.900	0.082	(0.063)	.191	0.039	(0.011)	.001	0.020	(0.010)	.055
153	Road injury	2.30%	2.98%	1.95%	2.03%	1.27%	0.017	(0.004)	.000	0.010	(0.005)	.034	0.016	(0.005)	.002	0.054	(0.027)	.047	0.028	(0.021)	.168
154	Poisonings	0.09%	0.10%	0.11%	0.03%	0.03%	0.004	(0.014)	.750	-0.006	(0.009)	.517	0.003	(0.025)	.891	0.010	(0.063)	.874	0.131	(0.035)	.000
155	Falls	2.25%	2.08%	1.74%	2.35%	4.54%	0.048	(0.005)	.000	0.035	(0.007)	.000	0.030	(0.007)	.000	0.065	(0.018)	.000	0.078	(0.017)	.000
156	Fire and heat injury	0.10%	0.09%	0.12%	0.04%	0.05%	0.019	(0.015)	.200	0.006	(0.016)	.724	0.015	(0.018)	.404	0.098	(0.081)	.228	0.109	(0.132)	.409
157	Drowning	0.00%	0.00%	0.01%	0.00%	0.01%	-0.013	(0.056)	.817				0.058	(0.001)	.000				-0.199	(0.004)	.000
158	Injuries from other mechanical forces	1.71%	2.41%	1.18%	1.30%	1.39%	0.026	(0.005)	.000	0.020	(0.005)	.000	0.014	(0.010)	.160	0.012	(0.015)	.432	0.087	(0.031)	.005
159	Other unintentional injuries	0.78%	0.83%	0.79%	0.59%	0.74%	0.020	(0.008)	.009	0.019	(0.013)	.127	0.004	(0.008)	.601	0.071	(0.043)	.096	0.042	(0.027)	.122
161	Self-harm	0.03%	0.01%	0.04%	0.04%	0.05%	0.096	(0.050)	.053	0.065	(0.059)	.272	0.051	(0.038)	.173	0.583	(0.166)	.000	-0.013	(0.094)	.891
162	Violence	0.09%	0.10%	0.09%	0.06%	0.07%	0.083	(0.029)	.004	0.085	(0.036)	.020	0.082	(0.056)	.146	0.131	(0.034)	.000	0.049	(0.106)	.646

Abbreviations: GHE, Global Health Estimates; KNHANES, Korea National Health and Nutrition Examination Survey; SE, standard error; OA, osteoarthritis.

^a^ If the two-tailed P value exceeded 0.10 (one-tailed P value > 0.05), the value of the disability weight was excluded from further analyses.

The modified disability-weights for visual impairments, stroke, osteoarthritis, back pain, and self-harm were distinctly larger than those for other conditions. When the disability-weight for each condition was compared between the age−sex groups, stroke, asthma, atopic dermatitis, rheumatoid arthritis, osteoarthritis, back pain, caries, periodontitis, falls, and other mechanical injuries had larger disability-weights in old than in young people. The disability-weights for iron-deficiency anemia and lung cancers were significant only in males. Diabetes had a relatively small disability-weight in young males, as compared to other age−sex groups.

### All-cause and condition-specific YLD

All-cause YLDs in 39044 thousand adults aged ≥20 years were 2165 thousand years. The combined YLDs (the sum of each condition-specific YLD) from noncommunicable diseases and injuries were similar to all-cause YLDs in old males, whereas those in young females accounted for about 50% of all-cause YLDs (Table 4).

**Table 4:** Condition-specific YLDs for noncommunicable diseases and injuries.

		YLDs of total^b^	Aggregate of YLDs^c^	YLDs	YLDs	YLDs	YLDs
GHE code		Total		Age 20−59 years		Age ≥ 60 years	
	KNHANES disease			Male	Female	Male	Female
	Population	39,044,074	39,044,074	15,737,018	15,041,580	3,572,458	4,693,018
0	All Causes	2,165,093	2,200,800	415,560	646,050	338,918	800,272
	Sum^a^	1,516,713	1,535,266	268,498	333,101	323,398	610,269
58	Iron-deficiency anemia						
	Mild	-7,928	-3,805	0	-3,805	0	0
	Moderate	0	5,451	1,275	0	4,176	0
	Severe	0	490	0	0	490	0
64	Stomach cancer	0	0	0	0	0	0
65	Colon cancers	0	2,413	0	0	2,413	0
66	Liver cancer	0	118	-412	530	0	0
68	Lung cancers	0	1,537	317	0	1,518	-299
70	Breast cancer	4,787	2,719	0	2,719	0	0
71	Cervix cancer	0	-2,036	0	0	0	-2,036
78	Other malignancy	0	0	0	0	0	0
80	Diabetes mellitus	68,662	72,413	17,280	13,140	21,686	20,306
83	Depression	103,900	102,035	26,881	48,610	6,688	19,856
86	Alcohol use disorders						
	Harmful drinking behavior	9,672	4,778	0	4,778	0	0
	Alcohol dependence	44,928	40,050	23,986	11,863	4,201	0
103	Glaucoma with visual impairment	0	696	696	0	0	0
104	Cataracts with visual impairment	29,424	29,664	2,459	1,651	14,206	11,348
105	Uncorrected refractive errors	17,174	14,598	8,515	6,083	0	0
106	Macular degeneration with visual impairment	5,322	3,374	-174	25	0	3,523
107	Diabetic retinopathy with visual impairment	2,068	1,104	90	0	1,013	0
108	Disabling hearing impairment	37,725	39,720	0	0	24,187	15,532
113	Ischemic heart disease	36,155	38,123	7,818	3,567	9,526	17,212
114	Stroke	61,295	64,633	12,254	4,807	26,332	21,240
118	COPD, age ≥40 years						
	Mild	0	0	0	0	0	0
	Moderate	0	3,605	0	3,605	0	0
	Severe	4,937	5,469	2,451	0	3,019	0
119	Asthma	37,361	37,473	5,430	7,208	7,796	17,040
122	Peptic ulcer, gastric or duodenal	31,451	32,141	7,862	6,870	4,481	12,929
123	Cirrhosis of the liver	0	0	0	0	0	0
127	Chronic kidney disease						
	Moderately increased risk	28,502	18,406	0	0	18,406	0
	High risk	9,799	0	0	0	0	0
	Very high risk	14,268	14,067	3,645	0	4,449	5,973
133	Atopic dermatitis	14,554	14,933	4,780	4,427	1,949	3,777
135	Rheumatoid arthritis	45,059	42,953	6,153	13,035	5,043	18,721
136	OA, age ≥50 years	199,650	200,751	6,320	21,937	29,856	142,638
138	Back pain						
	Back pain with radiographic OA, age ≥50 years	202,907	204,031	13,062	18,342	33,910	138,717
	Back pain without radiographic OA, age ≥50 years	177,350	178,841	17,603	43,149	38,853	79,236
	Back pain, age 20−49 years	107,488	107,348	38,820	68,528	0	0
148	Dental caries	42,614	46,429	9,441	13,383	9,958	13,647
149	Periodontal disease	68,075	69,465	11,025	12,615	14,295	31,530
150	Edentulism	23,579	21,729	0	0	12,200	9,530
153	Road injury	15,523	13,584	4,807	4,825	3,951	0
154	Poisonings	0	187	0	0	0	187
155	Falls	42,504	41,273	11,377	7,781	5,416	16,699
156	Fire and heat injury	0	0	0	0	0	0
157	Drowning	0	-24	0	58	0	-81
158	Injuries from other mechanical forces	17,266	13,106	7,432	0	0	5,675
159	Other unintentional injuries	6,091	1,500	0	0	1,500	0
161	Self-harm	1,149	746	0	0	746	0
162	Violence	2,818	1,572	1,296	0	276	0

Abbreviations: YLDs, years lived with disability; GHE, Global Health Estimates; KNHANES, Korea National Health and Nutrition Examination Survey; COPD, chronic obstructive pulmonary disease; OA, osteoarthritis

^a^ The combined YLDs from all the conditions investigated in this study.

^b^ The condition-specific YLDs calculated using the prevalence and disability weight obtained in the total population.

^c^ The aggregate of condition-specific YLDs of each age-sex group.

YLDs due to back pain and osteoarthritis were about 487745 and 199650, respectively, and were largest among all the condition-specific YLDs, particularly in old females. Additionally, depression, diabetes, stroke, and periodontitis had YLDs > 50000 years.

The aggregate of condition-specific YLDs for each age−sex group was similar to the condition-specific YLDs calculated using the prevalence and disability-weight determined in the total population.

### Comparison with WHO estimates

Table 5 shows the estimates converted to YLDs per 100000 people. The magnitude of the combined YLDs in our study was less than the WHO estimates. The ratio difference between our combined YLDs and the WHO YLDs was largest in young males, whereas it was minimal in old females. Many condition-specific YLDs in our study were less than those of the WHO estimates. The YLDs for iron-deficiency anemia, malignancies, diabetes, depression, alcohol-use disorders, visual/hearing impairment, ischemic heart disease, COPD, and injuries were substantially smaller than those of the GHE. However, the YLDs for stroke, peptic ulcer, osteoarthritis, back pain, caries, and periodontitis were larger than those of the WHO estimates. Particularly, the YLDs for back pain and osteoarthritis in old females were markedly larger than those of the WHO estimates.

**Table 5:** Comparisons of YLDs per 100000 people between the current study and the WHO’s global and regional estimates.

		Global	Study	ROK	Global		Study		ROK		Global		Study		ROK	
GHE code		Total			age 20 (or 15)^c^−59 years						age ≥ 60 years					
	GHE/KNHANES disease				Male	Female	Male	Female	Male	Female	Male	Female	Male	Female	Male	Female
	Population	100,000	100,000	100,000	100,000	100,000	100,000	100,000	100,000	100,000	100,000	100,000	100,000	100,000	100,000	100,000
0	All Causes	12,166	5,637	11,062	10,176	10,835	2,641	4,295	9,431	9,674	21,053	21,273	9,487	17,052	16,702	17,716
	Sum^a^	7,507	3,810		6,183	5,979	1,604	2,126			15,200	15,224	8,749	12,847		
	Sum^b^	6,232	3,192	5,914	5,527	5,345	1,354	1,901	5,498	4,549	10,611	10,459	6,583	10,912	9,735	9,395
58	Iron-deficiency anemia	341	5	61	196	510	8	-25	68	48	237	329	131	0	101	50
64	Stomach cancer	5	0	15	2	1	0	0	7	4	29	14	0	0	82	32
65	Colon and rectum cancers	11	6	22	4	3	0	0	9	6	63	46	68	0	105	65
66	Liver cancer	3	0	8	2	1	-3	4	6	1	15	7	0	0	43	15
68	Trachea, bronchus, lung cancers	7	4	10	3	1	2	0	4	2	51	18	42	-6	69	22
70	Breast cancer	18	7	23	0	18	0	18	0	27	0	124	0	0		112
71	Cervix uteri cancer	2	-5	2	0	4	0	0	0	4	0	8	0	-43		6
78	Other malignant neoplasms	12	0	16	7	6	0	0	7	9	50	32	0	0	56	45
80	Diabetes mellitus	420	185	629	289	286	110	87	441	448	1,121	1,143	607	433	1,345	1,424
83	Unipolar depressive disorders/Depression	1,315	261	832	1,020	1,662	171	323	652	1,042	883	1,465	187	423	583	939
86	Alcohol-use disorders	527	115	837	974	173	152	111	1,561	348	435	82	118	0	563	120
103	Glaucoma	24	2		5	6	4	0			110	141	0	0		
104	Cataracts	133	76		31	49	16	11			492	756	398	242		
105	Refractive errors/Uncorrected	253	37		104	129	54	40			902	1,066	0	0		
106	Macular degeneration	27	9		2	3	-1	0			124	194	0	75		
107	Other vision loss/Diabetic retinopathy	138	3		62	70	1	0			513	534	28	0		
108	Other hearing loss/Disabling hearing impairment	419	102		234	156	0	0			1,870	1,427	677	331		
113	Ischemic heart disease	177	98	186	104	82	50	24	107	96	703	562	267	367	564	506
114	Stroke	88	166	130	35	27	78	32	50	34	425	375	737	453	538	453
118	COPD/age ≥ 40 years	567	23	181	476	410	16	24	131	112	1,276	1,200	85	0	441	414
119	Asthma	156	96	132	145	161	35	48	121	128	163	186	218	363	156	170
122	Peptic ulcer disease	6	82	17	7	5	50	46	17	14	11	7	125	275	35	19
123	Cirrhosis of the liver	12	0	11	12	7	0	0	13	5	34	21	0	0	30	13
127	Kidney diseases/Chronic kidney disease	79	83	110	40	49	23	0	58	75	277	251	640	127	281	297
133	Skin diseases/Atopic dermatitis	231	38	232	192	216	30	29	187	210	380	370	55	80	368	370
135	Rheumatoid arthritis	75	110	150	23	86	39	87	50	171	77	276	141	399	130	456
136	Osteoarthritis/age ≥ 50 years	347	514	402	170	288	40	146	198	333	739	1,201	836	3,039	714	1,159
138	Back and neck pain/Back pain	974	1,256	1,070	906	859	442	864	964	964	1,530	1,413	2,037	4,644	1,532	1,484
148	Dental caries	79	119		82	85	60	89			52	53	279	291		
149	Periodontal disease	108	178		100	94	70	84			177	161	400	672		
150	Edentulism	95	56		36	42	0	0			350	434	341	203		
153	Road injury	252	35	208	337	169	31	32	273	147	354	145	111	0	307	113
154	Poisonings	8	0	1	10	5	0	0	2	1	17	7	0	4	2	1
155	Falls	380	106	450	286	185	72	52	344	220	1,347	998	152	356	1,352	975
156	Fire, heat, and hot substances	25	0	16	29	18	0	0	19	11	42	22	0	0	29	15
157	Drowning	5	0	2	6	3	0	0	2	1	13	6	0	-2	6	2
158+159	Other unintentional injuries including other forces	152	2	135	189	90	0	0	167	76	307	139	21	0	281	115
161	Self-harm	8	4	3	10	6	8	0	4	2	11	4	8	0	6	1
162	Interpersonal violence	30	0	21	52	14	0	0	37	10	22	6	0	0	15	4

Abbreviations: YLDs, years lived with disability; GHE, Global Health Estimates; KNHANES, Korea National Health and Nutrition Examination Survey; ROK, Republic of Korea; COPD, chronic obstructive pulmonary disease.

^a^ The combined YLDs from all the conditions investigated in this study.

^b^ The combined YLDs from all the investigated conditions available in regional estimates.

^c^ Age 20−59 years in this study or age 15−59 years in WHO’s global and regional estimates.

### Rank and percentage of YLD

The overall rank and the percentage of YLDs in this study differed from the WHO estimates (Table 6). Alcohol-use disorders, COPD, and injuries ranked lower in our study, and depression, diabetes, alcohol-use disorders, COPD, and injuries accounted for a reduced percentage of all-cause YLDs, than in the WHO estimates. In contrast, osteoarthritis, stroke, and peptic ulcer ranked higher and accounted for a greater percentage of all-cause YLDs than in the WHO estimates.

**Table 6:** The ranks of condition-specific YLDs and the percentage of all-cause YLDs accounted for by each condition-specific YLD

		% YLD	Rank		% YLD	Rank		% YLD	% YLD						% YLD					
		Global			Study	ROK			Global	Study	ROK	Global	Study	ROK	Global	Study	ROK	Global	Study	ROK
GHE code		Total							age 20 (or 15)^c^−59 years						age ≥ 60 years					
	GHE/KNHANES disease								Male			Female			Male			Female		
0	All Causes	100%			100%			100%	100%	100%	100%	100%	100%	100%	100%	100%	100%	100%	100%	100%
	Sum^a^	61.7%			67.6%				60.8%	60.8%		55.2%	49.5%		72.2%	92.2%		71.6%	75.3%	
	Sum^b^	51.2%			56.6%			53.5%	54.3%	51.3%	58.3%	49.3%	44.3%	47.0%	50.4%	69.4%	58.3%	49.2%	64.0%	53.0%
138	Back and neck pain/Back pain	8.0%	2	1	22.3%	1	1	9.7%	8.9%	16.7%	10.2%	7.9%	20.1%	10.0%	7.3%	21.5%	9.2%	6.6%	27.2%	8.4%
136	Osteoarthritis/age ≥ 50 years	2.9%	8	2	9.1%	2	6	3.6%	1.7%	1.5%	2.1%	2.7%	3.4%	3.4%	3.5%	8.8%	4.3%	5.6%	17.8%	6.5%
83	Unipolar depressive disorders/Depression	10.8%	1	3	4.6%	3	3	7.5%	10.0%	6.5%	6.9%	15.3%	7.5%	10.8%	4.2%	2.0%	3.5%	6.9%	2.5%	5.3%
80	Diabetes mellitus	3.4%	5	4	3.3%	4	4	5.7%	2.8%	4.2%	4.7%	2.6%	2.0%	4.6%	5.3%	6.4%	8.1%	5.4%	2.5%	8.0%
149	Periodontal disease	0.9%	18	5	3.2%				1.0%	2.7%		0.9%	2.0%		0.8%	4.2%		0.8%	3.9%	
114	Stroke	0.7%	20	6	2.9%	5	14	1.2%	0.3%	2.9%	0.5%	0.2%	0.7%	0.4%	2.0%	7.8%	3.2%	1.8%	2.7%	2.6%
148	Dental caries	0.6%	21	7	2.1%				0.8%	2.3%		0.8%	2.1%		0.2%	2.9%		0.2%	1.7%	
86	Alcohol-use disorders	4.3%	4	8	2.0%	6	2	7.6%	9.6%	5.8%	16.5%	1.6%	2.6%	3.6%	2.1%	1.2%	3.4%	0.4%	0.0%	0.7%
135	Rheumatoid arthritis	0.6%	23	9	2.0%	7	11	1.4%	0.2%	1.5%	0.5%	0.8%	2.0%	1.8%	0.4%	1.5%	0.8%	1.3%	2.3%	2.6%
155	Falls	3.1%	7	10	1.9%	8	5	4.1%	2.8%	2.7%	3.6%	1.7%	1.2%	2.3%	6.4%	1.6%	8.1%	4.7%	2.1%	5.5%
108	Other hearing loss/Disabling hearing impairment	3.4%	6	11	1.8%				2.3%	0.0%		1.4%	0.0%		8.9%	7.1%		6.7%	1.9%	
113	Ischemic heart disease	1.5%	13	12	1.7%	9	9	1.7%	1.0%	1.9%	1.1%	0.8%	0.6%	1.0%	3.3%	2.8%	3.4%	2.6%	2.2%	2.9%
119	Asthma	1.3%	14	13	1.7%	10	13	1.2%	1.4%	1.3%	1.3%	1.5%	1.1%	1.3%	0.8%	2.3%	0.9%	0.9%	2.1%	1.0%
127	Kidney diseases/Chronic kidney disease	0.6%	22	14	1.5%	11	15	1.0%	0.4%	0.9%	0.6%	0.5%	0.0%	0.8%	1.3%	6.7%	1.7%	1.2%	0.7%	1.7%
122	Peptic ulcer disease	0.1%	35	15	1.5%	12	20	0.2%	0.1%	1.9%	0.2%	0.0%	1.1%	0.1%	0.1%	1.3%	0.2%	0.0%	1.6%	0.1%
104	Cataracts	1.1%	17	16	1.3%				0.3%	0.6%		0.5%	0.3%		2.3%	4.2%		3.6%	1.4%	
150	Edentulism	0.8%	19	17	1.0%				0.4%	0.0%		0.4%	0.0%		1.7%	3.6%		2.0%	1.2%	
133	Skin diseases/Atopic dermatitis	1.9%	12	18	0.7%	13	7	2.1%	1.9%	1.2%	2.0%	2.0%	0.7%	2.2%	1.8%	0.6%	2.2%	1.7%	0.5%	2.1%
105	Refractive errors/Uncorrected	2.1%	10	19	0.7%				1.0%	2.0%		1.2%	0.9%		4.3%	0.0%		5.0%	0.0%	
153	Road injury	2.1%	11	20	0.6%	14	8	1.9%	3.3%	1.2%	2.9%	1.6%	0.7%	1.5%	1.7%	1.2%	1.8%	0.7%	0.0%	0.6%
118	COPD/age ≥ 40 years	4.7%	3	21	0.4%	15	10	1.6%	4.7%	0.6%	1.4%	3.8%	0.6%	1.2%	6.1%	0.9%	2.6%	5.6%	0.0%	2.3%
106	Macular degeneration	0.2%	25	22	0.2%				0.0%	0.0%		0.0%	0.0%		0.6%	0.0%		0.9%	0.4%	
70	Breast cancer	0.1%	28	23	0.1%	16	17	0.2%	0.0%	0.0%	0.0%	0.2%	0.4%	0.3%	0.0%	0.0%	0.0%	0.6%	0.0%	0.6%
65	Colon and rectum cancers	0.1%	31	24	0.1%	17	18	0.2%	0.0%	0.0%	0.1%	0.0%	0.0%	0.1%	0.3%	0.7%	0.6%	0.2%	0.0%	0.4%
58	Iron-deficiency anemia	2.8%	9	25	0.1%	18	16	0.6%	1.9%	0.3%	0.7%	4.7%	-0.6%	0.5%	1.1%	1.4%	0.6%	1.5%	0.0%	0.3%
162	Interpersonal violence	0.2%	24	26	0.1%	19	19	0.2%	0.5%	0.3%	0.4%	0.1%	0.0%	0.1%	0.1%	0.1%	0.1%	0.0%	0.0%	0.0%
68	Trachea, bronchus, lung cancers	0.1%	34	27	0.1%	20	25	0.1%	0.0%	0.1%	0.0%	0.0%	0.0%	0.0%	0.2%	0.4%	0.4%	0.1%	0.0%	0.1%
107	Other vision loss/Diabetic retinopathy	1.1%	16	28	0.1%				0.6%	0.0%		0.6%	0.0%		2.4%	0.3%		2.5%	0.0%	
161	Self-harm	0.1%	33	29	0.0%	21	27	0.0%	0.1%	0.0%	0.0%	0.1%	0.0%	0.0%	0.1%	0.2%	0.0%	0.0%	0.0%	0.0%
103	Glaucoma	0.2%	27	30	0.0%				0.0%	0.2%		0.1%	0.0%		0.5%	0.0%		0.7%	0.0%	
154	Poisonings	0.1%	32	31	0.0%	22	30	0.0%	0.1%	0.0%	0.0%	0.0%	0.0%	0.0%	0.1%	0.0%	0.0%	0.0%	0.0%	0.0%
66	Liver cancer	0.0%	38	32	0.0%	23	26	0.1%	0.0%	-0.1%	0.1%	0.0%	0.1%	0.0%	0.1%	0.0%	0.3%	0.0%	0.0%	0.1%
64	Stomach cancer	0.0%	37	33	0.0%	24	23	0.1%	0.0%	0.0%	0.1%	0.0%	0.0%	0.0%	0.1%	0.0%	0.5%	0.1%	0.0%	0.2%
78	Other malignant neoplasms	0.1%	30	34	0.0%	25	21	0.1%	0.1%	0.0%	0.1%	0.1%	0.0%	0.1%	0.2%	0.0%	0.3%	0.2%	0.0%	0.3%
123	Cirrhosis of the liver	0.1%	29	35	0.0%	26	24	0.1%	0.1%	0.0%	0.1%	0.1%	0.0%	0.0%	0.2%	0.0%	0.2%	0.1%	0.0%	0.1%
156	Fire, heat, and hot substances	0.2%	26	36	0.0%	27	22	0.1%	0.3%	0.0%	0.2%	0.2%	0.0%	0.1%	0.2%	0.0%	0.2%	0.1%	0.0%	0.1%
158+159	Other unintentional injuries including other forces	1.2%	15	37	0.0%	28	12	1.2%	1.9%	0.0%	1.8%	0.8%	0.0%	0.8%	1.5%	0.0%	1.7%	0.7%	0.0%	0.7%
157	Drowning	0.0%	36	38	0.0%	29	29	0.0%	0.1%	0.0%	0.0%	0.0%	0.0%	0.0%	0.1%	0.0%	0.0%	0.0%	0.0%	0.0%
71	Cervix uteri cancer	0.0%	39	39	-0.1%	30	28	0.0%	0.0%	0.0%	0.0%	0.0%	0.0%	0.0%	0.0%	0.0%	0.0%	0.0%	-0.3%	0.0%

Abbreviations: YLDs, years lived with disability; GHE, Global Health Estimates; KNHANES, Korea National Health and Nutrition Examination Survey; ROK, Republic of Korea; COPD, chronic obstructive pulmonary disease.

^a^ The combined YLDs from all the conditions investigated in this study.

^b^ The combined YLDs from all the investigated conditions available in regional estimates.

^c^ Age 20−59 years in this study or age 15−59 years in WHO’s global and regional estimates.

The five leading causes of YLDs were back pain, depression, alcohol-use disorders, diabetes, and stroke, in young males, whereas these were back pain, depression, osteoarthritis, alcohol-use disorders, and caries in young females. Back pain, osteoarthritis, stroke, hearing impairment, and CKD were the top-five YLD causes in old males, while back pain, osteoarthritis, periodontitis, stroke and diabetes ranked highest in old females.

## Discussion

We here readily estimated population-level YLDs for noncommunicable diseases and injuries using a prevalence-based approach, in which we measured the disability-weight in subjects with specific disease or injury. To the best of our knowledge, this is the first report to date estimating YLDs for numerous conditions using prevalence rates and disability-weights both measured in a representative sample. This new approach revealed that the increase of combined YLDs from noncommunicable diseases with ageing, which is more distinct in females, was mostly due to the exceptionally large YLDs ascribed to back pain and osteoarthritis, particularly in old females.

Back pain and osteoarthritis were very common, particularly in old females, and the disability-weights for those diseases were exceptionally large. The disability-weights were also large for visual impairments (except in uncorrected refractive errors) and stroke, but the prevalence rates of these conditions were much lower than those of back pain or osteoarthritis. Diabetes, alcohol-use disorders, hearing impairment, COPD, CKD, and periodontitis were also very common, but the disability-weights were not as large as those for back pain or osteoarthritis.

Our study findings differed somewhat from those of the GHE. The condition-specific YLDs for many diseases and injuries (except for back pain, osteoarthritis, periodontitis, stroke, CKD, caries, or peptic ulcer) were lower than those of the GHE, and the combined YLDs for all conditions in most age−sex groups (except in old females) were also lower than those of the WHO estimates. In contrast to the WHO estimates, the combined YLDs in our study differed markedly between males and females. The differences in male and female health-related quality of life have been observed not only in our study of the Korean population, but also in studies of other ethnic populations [20–22].

The GBD 2010 used lay descriptions of the symptoms and dysfunctions resulting from diseases or injuries to estimate disability-weights for those conditions, and obtained highly consistent values across surveys performed in diverse communities [9]. Although these brief descriptions were straightforward, they may fail to reflect different manifestations of any given disease in terms of severity, treatment, or environment. A number of studies have evaluated DALYs or YLDs for various diseases [23–27], and most have used health state descriptions to estimate disability-weights, following the GBD and WHO method. It is doubtful that the abstract values of disability-weights obtained using descriptions that assume typical manifestations of diseases could reflect a real-life health state. Previous YLDs differed markedly across previous studies, particularly in the case of mild disease [28]. The manifestations of disease may vary from asymptomatic to apparently symptomatic depending on individual conditions and environments. In general, mild disease states, with no or vague symptoms, are common, whereas severe states, with apparent symptoms, are relatively uncommon. If a disability-weight for a severe, uncommon disease state is used to estimate the YLDs of a mild common disease state, the YLDs may be erroneously overestimated. The lack of population information on the severity distribution of most conditions may frequently lead to mismatch errors between prevalence rates and disability-weights in the calculation of YLDs. These types of mismatch errors can be avoided by measuring both values from a single study sample.

A few studies have assessed DALYs or YLDs using a disability-weight that was directly measured in patients with specific diseases or injuries [5,29–33]. However, most of them evaluated YLDs for only one or two conditions, and even did so without reasonable reference groups. The aggregate of separate estimations of YLDs for various diseases, without relevant mutual exclusions between disease categories, may result in overestimation of total YLDs, due to the duplications of the YLDs. The GHE list, from which we identified specific diseases and injuries for this study, may provide mutually exclusive and aggregative categories. In our study, the disability-weight was measured in subjects with a specific disease or injury; both the prevalence and disability-weight were measured in a representative sample, and the YLDs from dozens of conditions in the GHE list were estimated from the same source. Thus, our estimates of the condition-specific and combined YLDs were more likely to reflect the real health state of the population and to overcome erroneous estimation due to mismatch errors or duplicated counts.

There was a marked, age-related increase in YLDs ascribed to osteoarthritis and back pain, with a notable difference between males and females. The YLDs from osteoarthritis and back pain were exceptionally large, particularly for older females. The combined YLDs from both these conditions accounted for 31.5% of all-cause YLDs in adults aged ≥ 20 years. In the Global Burden of Disease (GBD) 2010 and WHO GHE, back pain and osteoarthritis also ranked highest. However, the YLDs from those were not as marked as in our estimates, and did not differ between the sexes. Our finding of the sex differences in YLDs for back pain and osteoarthritis could possibly explain the worse health-related quality of life in females, which has also been demonstrated in previous studies performed in other ethnic groups [20–22]. For osteoarthritis, the differences in YLDs for the two sexes resulted mainly from the difference in prevalence rates. The prevalence of osteoarthritis in females was markedly higher than that in males, while the disability-weight was similar between the sexes. As we confirmed osteoarthritis from radiographs as well as from symptoms, our prevalence estimate is reliable.

On the other hand, back pain, the single highest-ranked condition, was common in young people as well as in old people. However, the disability-weight from back pain was relatively small in young people as compared to old people. Back pain has diverse causes, including osteoarthritis, herniated disks, instability, spinal stenosis, and the sequelae of spine surgery, and is most frequently diagnosed as “nonspecific back pain” [34]. In our study, in half of old people with back pain, this disorder was accompanied by radiographic osteoarthritis, but this accompanying rate was sharply decreased in young people. Further research is urgently needed to define the broad category of “back pain” better.

Additionally, the YLD estimates of our study incorporate the effect of current treatments as well as the severity of the disorder itself. This point should be considered when interpreting our results. Back pain and osteoarthritis should receive greater emphasis in terms of disability, particularly in older women. However, preventative strategies or brief supportive care, rather than traditional or specialized treatments, may be more effective in reducing osteoarthritis and back pain [35,36].

Diabetes is another important cause of disability. The YLDs from diabetes were 72000 years and accounted for 3.3% of all-cause YLDs. Diabetes is a common disease that has various complications. The GBD 2010 and WHO GHE used discrete disability-weights according to the complication of diabetes (uncomplicated, diabetic foot, and diabetic neuropathy). However, it may be difficult to establish the distribution of complications at the age, sex, and regional level. In the KNHANES sample, the microvascular complication rates of diabetes differed according to age and sex. Diabetic retinopathy/nephropathy (urine albumin-to-creatinine ratio ≥ 30 mg/g) was observed in 13.7%/19.1%, 13.9%/19.7%, 20.2/29.7%, and 19.5/24.7% of the young-male, young-female, old-male, and old-female diabetics, respectively. In our study, the disability-weight ascribed to diabetes was 0.015, 0.021, 0.026, and 0.021, in the young males, young females, old males, and old females, respectively. We believe that our data represent more reliable disability-weights and YLDs for diabetes at the age and sex level.

Depression and alcohol-use disorders are well known to be major contributors to disability. The YLDs from depression/alcohol-use disorders accounted for 4.6%/2.0% of all-cause YLDs, respectively, and the values were notably different from the WHO’s global (10.8%/4.3%) and regional (7.5%/7.6%) estimates. This large difference was caused by the marked differences in the disability-weights between the GHE and our study. The GHE disability-weights for major depression and alcohol-use disorders ranged from 0.159 to 0.655, but the overall disability-weights for depression and alcohol-use disorders were 0.072 and 0.017, respectively, in our study. The disability due to these mental and behavioral disorders could easily be affected by the social or cultural environment. Moreover, the severity of the disorders could be differently regarded by the patients themselves and by those around them. We obtained disability-weights from a self-reported questionnaire (EQ-5D), using the Korean value set that was established based on a representative national sample. The disability-weights and YLDs of our research therefore incorporate cultural effects and self-assessments.

The overall YLDs from visual impairment (including uncorrected refractive errors) accounted for 2.3% of all-cause YLDs. In adults aged ≥ 20 years, the overall prevalence of visual impairments (including blindness) with best-corrected visual acuity < 8/16 in the better eye was 1.58%, and the prevalence of uncorrected refractive errors was 3.85%. When each cause was calculated as a percentage of total causes of visual impairment (excluding uncorrected refractive errors), the causes were cataract (62.0%), glaucoma (10.1%), age-related macular degeneration (8.3%), diabetic retinopathy (3.6%), and undetermined causes (16.0%). Visual impairment from undetermined causes did not decrease the EQ-5D index scores in our study (data not shown), although that was the largest global cause of YLDs ascribed to visual impairment according to the GHE. Cataracts, uncorrected refractive errors, and macular degeneration were the top three contributors to YLDs due to visual impairment. A total of 29487 adults underwent ophthalmologic examinations in the KNHANES from July 2008 to December 2012, and trained medical staff and ophthalmologists conducted the examinations using standardized equipment and protocols. Our results may be helpful for the estimation of the global or regional burden of visual impairment.

The combined YLDs from all injuries accounted for 2.6% of all-cause YLDs. Falls and road injury accounted for 95.7% of the total YLDs from all injuries. Falls and road injury were common in old women and in young men, respectively. Although the sequelae of injuries may have a wide spectrum of severity, it may be very hard to identify the severity distribution of sequelae at the population level. Previous studies performed in European countries have suggested that injuries are main contributors to YLDs, as well as years of life lost (YLLs) [33,37]. Those studies analyzed the data based on the disability-weights obtained from patients in hospital or emergency settings. The disability-weights obtained from hospitalized patients or emergency department attendances would reflect disability for severe injuries, but would not represent disability for injuries of various states. As mentioned earlier, if the disability-weight for severe injuries was applied to a mild state, the YLDs may be overestimated. In contrast, our research was based on the KNHANES, which involved non-institutionalized civilians only, and investigated recent (within 1 year) injuries. People with severe conditions or lifelong sequelae were more likely to be excluded, and our results may underestimate the YLDs from injuries.

Stroke/ischemic heart disease accounted for 166/98 YLDs per 100000 adults and 2.9%/1.7% of all-cause YLDs in our study, whereas they accounted for 130/186 YLDs per 100000 adults and 1.2%/1.7% of all-cause YLDs in the WHO’s regional estimates. Percutaneous coronary intervention is well known to provide a benefit in terms of quality of life in patients with ischemic heart disease [38]. The existence of effective treatment may result in the contrasting YLDs between diseases. Disability due to hearing impairment could also be affected by the availability of medical resources. A substantial improvement in the mental health quality of life after cochlear implant or hearing aid use has been reported in patients with hearing impairments [39]. It is quite possible that the YLDs from hearing impairment are smaller (our estimates) than expected (WHO’s global estimates) with the aid of these modalities. The YLDs from chronic obstructive pulmonary disease was less than the WHO’s global or regional estimates. In old females the degree of decreased forced expiratory volume in 1 second did not correlate with the severity of disability. It remains possible that the assessment of chronic obstructive pulmonary disease based only on the results of a pulmonary function test cannot readily estimate the severity in old females. Chronic kidney disease also contributes to YLDs. Interestingly, chronic kidney disease with moderately increased risk significantly contributed to YLDs only in old men, whereas a more advanced state (with high risk) did not in old men. The potential overestimation of the glomerular filtration rate in the case of muscle wasting, a common problem in the elderly suffering from kidney disease, may influence the association between disability and estimated kidney function in old people. Peptic ulcer accounted for 1.5% of all-cause YLDs, and the value was larger than the WHO’s global (0.1%) and regional (0.2%) estimates. We believe that the relatively larger YLDs due to peptic ulcer reflect the regional differences in the characteristics of peptic ulcers [40]. Oral health disorders (dental caries, periodontitis, and edentulism) were important contributors to YLDs. Oral health disorder is a preventable disease and is related to general hygiene. It is therefore necessary to emphasize the importance of oral hygiene. The magnitude of YLDs due to cancers was relatively small. Malignancy is a well-known major contributor to YLLs, but the YLDs due to malignancy is not thought be large. Nevertheless, colorectal and lung cancers in old males and breast cancer in females were significant contributors to YLDs. Interestingly, iron-deficiency anemia, contributed to YLDs only in males. The causes of iron deficiency, physical activity, or comorbidities may be involved in the association between iron-deficiency anemia and YLDs. Overall, these findings suggest that multifactorial processes are involved in the determination of health-related quality of life in the general population.

Taken together, the YLDs estimated in this study differed somewhat from those of the GBD 2010 and WHO GHE. The differences might arise from three different sources. First, we estimated the prevalence rates of 40 conditions in a single representative sample, whereas those of the GBD and GHE were obtained from various sources. Additionally, we confirmed many diseases by objective physical/laboratory findings along with patient’s symptoms, to obtain more precise estimates of disease prevalence. Our estimates might provide a more consistent and reliable source for comparing disease burdens among numerous conditions. Second, we generated disability-weights from the EQ-5D index directly measured in a large sample from the KNHANES, whereas the GBD 2010 measured disability-weights using lay health-state descriptions, which could not reflect various manifestations of the same disease particularly in terms of severity. Additionally, we computed disability-weights separately in each age and sex category (young males, young females, old males, and old females). Disease burdens estimated in our study might incorporate age-specific effects as well as the severity of the disorder. Finally, we could overcome erroneous estimation of YLDs from mismatch errors between prevalence rates and disability-weights through measuring them in a single study sample. The GBD rankings are based on epidemiological data that may not be sufficiently robust for the calculation of the YLDs; the lack of reliable information on severity distributions may lead to mismatch errors in the calculation of YLDs. We believe that our YLD estimates are more likely to reflect real health states of the population.

On the other hand, the use of computed disability-weights from people having health conditions is not necessary an advantage compared to the weights that use lay health-state descriptions as a basis. Since persons with health conditions tend to underestimate their level of disability, the use of weights based on lay descriptions is the most conservative approach. In addition, disability-weights based on lay descriptions have been preferred for the estimation of disease burdens because they take into account the opinion of the general population.

There are several points to consider when interpreting our results. First, we did not investigate all the causes of YLD in the GHE list. In addition to the investigated diseases, anxiety disorders, migraine, schizophrenia, drug-use disorders, and gynecologic disorders are important contributors to the global disease burden. We could not include such conditions, due to the lack of relevant information in the KNHANES data. However, the combined YLDs from the conditions included in this study accounted for 61.7% of all-cause YLDs in the WHO’s global estimates. Additionally, we investigated visual/hearing impairments and oral health disorders, which were not included in the WHO’s regional estimates. Second, certain diseases were defined by a physician-based diagnosis of the disease, while many other diseases were confirmed by physical/laboratory examinations, in conjunction with the patient’s history. The prevalence of a diagnosed disease could different from the true prevalence. Some diagnoses may have been incorrect or missed. Third, as the survey enrolled non-institutionalized individuals who volunteered to participate, persons with severe conditions were more likely to be excluded. Thus, the estimates would have been underestimated in the KNHANES. Finally, the present study included subjects who resided in Korea, and the disability-weights were generated based on the EQ5D index scores calculated using the Korean value set, which were closer to values of the Japanese study than those of studies in western countries [12]. Since there is likely to be regional or ethnic differences in disease burdens, it is difficult to draw general conclusions applicable to the global population. Nevertheless, the values of prevalence, disability-weight, and YLDs determined in our study may be helpful in estimating non-fatal burdens of diseases in East Asia, where populations with similar ethnic and cultural backgrounds reside.

## Conclusions

This relatively simple, prevalence-based approach, using a population-representative survey, could readily estimate YLDs reflecting the real health state of the general population. The results of this study may form the basis for population-level strategies to prevent age-related worsening of disability, which is more severe in females.
